# Prevalence of multimorbidity in older adults in São Paulo, Brazil: a study with ISA-Capital

**DOI:** 10.11606/s1518-8787.2022056004252

**Published:** 2022-07-21

**Authors:** Kaio Keomma, Aylene Bousquat, Chester Luiz Galvão César

**Affiliations:** I Universidade de São Paulo Faculdade de Saúde Pública Programa de Pós-Graduação em Saúde Pública São Paulo SP Brasil Universidade de São Paulo . Faculdade de Saúde Pública . Programa de Pós-Graduação em Saúde Pública . São Paulo , SP , Brasil; II Universidade de São Paulo Faculdade de Saúde Pública Departamento de Política, Gestão e Saúde São Paulo SP Brasil Universidade de São Paulo . Faculdade de Saúde Pública . Departamento de Política, Gestão e Saúde . São Paulo , SP , Brasil; III Universidade de São Paulo Faculdade de Saúde Pública Departamento de Epidemiologia São Paulo SP Brasil Universidade de São Paulo . Faculdade de Saúde Pública . Departamento de Epidemiologia . São Paulo , SP , Brasil

**Keywords:** Aged. Multimorbidity, Prevalence, Risk Factors, Health Surveys

## Abstract

**OBJECTIVE:**

To estimate the prevalence of multimorbidity in older adults in São Paulo, Brazil.

**METHODS:**

A cross-sectional study based on the 2015 ISA-Capital population-based survey, with a subsample of 1,019 older adults aged ≥ 60 years old. Multimorbidity was categorized considering two or more chronic diseases, based on a previously defined list. The data were analyzed in univariate and multiple models with Poisson regression.

**RESULTS:**

The prevalence of multimorbidity was 40% (95%CI: 36.6–43.8), being higher in women (PR _a_ = 1.95 [compared to men]; 95%CI: 1.58–2.40), in individuals aged ≥ 75 years old (PR _a_ = 1.25 [compared to individuals aged ≥ 60 to 64 years old]; 95%CI: 1.01–1.60), in Black people (PR _a_ = 1.28 [compared to White people]; 95%CI: 1.04–1.59), in high-income people (PR _a_ = 1.27 [compared to low income]; 95%CI: 1.09–1.50) and in former smokers (PR _a_ = 1.30 [compared to those who never smoked]; 95%CI: 1.05–1.60), and lower in smokers (PR _a_ = 0.72 [compared to those who never smoked]; 95%CI: 1.09–1.50).

**CONCLUSION:**

The prevalence of multimorbidity was lower than that reported in most of the reviewed studies, but consistently associated with gender, age, race/skin color, smoking habit and socioeconomic status. The standardization of conceptual and methodological criteria for estimation is a challenge to relieve problems in the planning and management of health care systems for older populations.

## INTRODUCTION

Multimorbidity is the coexistence of multiple chronic diseases in an individual ^1^ . It usually involves two conditions ^2^ , but without consensus on its conceptualization or on the most appropriate methods to estimate it, from simple accounting per individual to sophisticated classification systems to measure morbidity load ^3^ , which widely varies estimates and associations ^4^ .

Multimorbidity is a worldwide health problem ^5^ . Our findings show that its prevalence affects more than half of the older adults ^6^ , which is higher in women ^7^ , poorer individuals and residents of urban areas ^8^ , Black people ^9^ and former smokers ^10^ , with great impact on mortality ^11 , 12^ . Covid-19 (SARS-Cov-2) exposed this, in which up to 72% chronic and multimorbidity patients represented the patients admitted to intensive care units in some locations ^13^ . These individuals were more vulnerable during the pandemic ^14^ and the unawareness of this indicator in many locations, the greater alignment of services to acute conditions, restriction to appointments, elective procedures, among others, can contribute to this.

Multimorbidity has affected indicators such as successive hospitalizations, extended hospitalizations, polypharmacy, simultaneous use of many services of different levels of technological density, care cost and the coordination of care, because it requires many people and services for health care ^15^ . Understanding these and other issues is essential to the health care system and necessarily involves knowledge about their magnitude and distribution. However, studies estimating the prevalence of multimorbidity are incipient in Brazil, especially with representative samples ^5 , 8 , 16^ .

This study aims to contribute to fill part of the gap of studies on population-based multimorbidity in the Brazilian literature, supporting criticism and debate by making evidence available to researchers, managers and the society. Therefore, we aimed to analyze the prevalence of multimorbidity in older adults in the city of São Paulo, Brazil.

## METHODS

This cross-sectional study was conducted with 1,019 older adults aged ≥ 60 years old participating in the ISA-Capital health survey, which is a population-based survey in the city of São Paulo, Brazil, conducted in 2015.

The sampling of the original study occurred by complex and probabilistic methodology, with drawings of census and household sectors. The geographical and demographic domains were considered, including, respectively, the regional health coordinators (Midwest, East, North, Southeast and South) and different age groups (adolescents 12 to 19 years old, adult men 20 to 59 years old, adult women 20 to 59 years old and men and women aged ≥ 60 years old). The algebraic expression $n=p.\left(1-p\right)/{\left(d/z\right)}^{2}deff$ . *deff* was considered, in which: *n* is the sample size, *p* is the parameter to be estimated, z = 1.96 is the value in the reduced normal curve referring to 95% confidence level of the confidence intervals, d is error and deff is the effect of the design ^20^ .

The participants were recruited considering the inclusion criteria: being within the included age groups, living in the urban area and in private households permanently. Homeless people or residents of institutions were excluded. The non-response rate due to vacant, closed households, with refusal or with resident unable to respond, was observed. The inclusion of a sample greater than necessary was planned to reach a minimal of interviews in case of losses. Thus, the desired number of interviews was reached ^20^ .

The variables considered included: gender (male/female); age in full years (60–64, 65–69, 70–74, ≥ 75 years old); race/skin color (White, Black, Mixed-race and Asian); consumer price index, which was considered a socioeconomic proxy, based on the *Critério de Classificação Econômica Brasil* (CCEB - Brazilian Economic Classification Criterion), dichotomized in high income categories (for strata originally classified as A1, A2 and B1) and low income (for B2, C1 and C2, D, E strata); smoking habit (never smoked, former smoker, smoker); and multimorbidity, measured by counting two or more self-reported morbidities from a previously elaborated list ^7^ .

A total of 10 chronic diseases from the baseline study were considered to compose multimorbidity: systemic arterial hypertension, type 2 diabetes mellitus, osteoporosis, arthritis/rheumatism, stroke, acute myocardial infarction, chronic obstructive pulmonary disease (COPD), neoplasia, Alzheimer’s and Parkinson’s. Acute conditions, risk factors or phenotypic characteristics such as high cholesterol, back pain, rhinitis, sinusitis and tendinitis were excluded. Although this variable has more than one definition that can be accepted ^21^ , especially regarding number and included conditions, the strategy used agrees with the reviewed literature because it includes many body systems and clusters around cardiovascular, metabolic and musculoskeletal disorders ^7^ .

Data analysis was operationalized with the Survey module of the Stata program, version 14.0, for data from complex samples. The prevalence of general multimorbidity and according to gender, age, race/skin color, smoking habit and consumer price index, including the calculation of proportion of occurrence (%), 95% confidence interval (95%CI) and p-value by Pearson’s chi-square test, were estimated. Univariate (unadjusted) and multiple (adjusted) models were proposed to evaluate the variation in the prevalence of multimorbidity, variable outcome and other independent variables, with Poisson regression. Crude (PR) and adjusted (PR _a_ ) prevalence ratios and 95%CI were obtained. Associations with 95%CI without including nullity (PR = 1.00) and p < 0.05 were considered statistically significant.

All analyses performed – obtaining proportions, confidence intervals and tests – were considered with the participants’ final weight for statistical inference, calculated based on: (1) Design weight, which considers the sampling fractions of the two drawing stages, the census and household sectors; (2) Adjustment of non-response, which considers the observed response rates; (3) Post-stratification, which adjusts the sample distribution by gender, age and residence, according to the population distribution of the study scenario estimated for the research year.

The ISA-SP was approved by the ethics and research committee of the Faculdade de Saúde Pública of Universidade de São Paulo, and all ethical and legal precepts were observed.

## RESULTS

The participants were mainly women (59.7%), the mean age was 67.7 years old (DP = 7.7), one quarter were ≥ 75 years old (25.2%), most of them reported White race/skin color (59.8%), low income (81%) and never having smoked (63.4%) ( Table 1 ).

**Table 1:** Characterization of the participants in the study. São Paulo, Brazil, 2015.

Variables	n (%) ^a^
Gender	
Female	632 (59.7)
Male	387 (40.3)
Age	
60–64 years old	310 (30.9)
65–69 years old	263 (26.2)
70–74 years old	185 (17.5)
≥ 75 years old	261 (25.2)
Race/Skin color	
Black	134 (11.9)
Mixed-race	263 (23.7)
White	571 (59.8)
Asian	39 (4.3)
Smoking habit	
Former smoker	249 (24.5)
Never smoked	644 (63.4)
Smoker	124 (12.3)
Consumer price index	
A (high income)	176 (23.6)
B (low income)	749 (76.4)

^a^ Number of respondents without weighting.

The overall prevalence of multimorbidity was estimated at 40% (95%CI: 36.6–43.8), being significantly higher among women (49.1%; 95%CI: 44.7–54.0; p < 0.001), those aged ≥ 75 years old (45.1%; 95%CI: 37.6–54.0; p = 0.036) and in former smokers (44.3%; 95%CI: 38.2–51.5; p = 0.002) ( Table 2 ).

**Table 2:** Prevalence of multimorbidity in older adults, according to independent variables. São Paulo, Brazil, 2015.

Variables	(95%CI)	p
Gender		**< 0.001**
Female	49.1 (44.7–54.0)	
Male	26.5 (22.1–31.8)	
Age		**0.036**
60–64 years old	34.4 (29.0–40.9)	
65–69 years old	40.5 (34.7–47.2)	
70–74 years old	42.0 (34.6–51.1)	
≥ 75 years old	45.1 (37.6 – 54.0)	
Race/Skin color		0.591
Black	46.3 (38.5–55.8)	
Mixed-race	41.3 (34.8–48.9)	
White	38.5 (33.9–43.6)	
Asian	37.8 (24.0–59.4)	
Smoking habit		**0.002**
Former smoker	44.3 (38.2–51.5)	
Never smoked	41.3 (36.9–46.1)	
Smoker	25.6 (18.9–34.4)	
Consumer price index		0.149
High income	43.5 (36.8–51.4)	
Low income	38.1 (34.5–42.2)	

95%CI: 95% confidence interval.

The univariate analysis, which analyzed multimorbidity without adjusting the independent variables, showed that being a woman (PR = 1.85 [compared to men]; 95%CI: 1.52–2.25), ≥ 75 years old (PR = 1.30 [compared to individuals aged ≥ 60 to 64 years old]; 95%CI: 1.01–2.68) and being a smoker (PR = 0.62; 95%CI: 0.45–0.84) were significantly associated. Except for this last condition that reduced multimorbidity by 38%, the first two increased it by 85% and 30%, respectively ( Table 3 ).

**Table 3:** Prevalence ratio of multimorbidity in older adults, according to independent variables in an unadjusted univariate model. São Paulo, Brazil, 2015.

Variables	Univariate analysis		
	PR	95%CI	p
Gender (ref. male)			
Female	1.85	1.52–2.25	**< 0.001**
Age (ref. 60–64 years old)			
65–69 years old	1.18	0.94–1.46	0.147
70–74 years old	1.22	0.93–1.59	0.137
≥ 75 years old	1.30	1.01–1.68	**0.035**
Race/Skin color (ref. White)			
Asian	0.98	0.63–1.53	0.938
Mixed-race	1.07	0.87–1.32	0.519
Black	1.20	0.97–1.48	**0.078**
Smoking habit (ref. never smoked)			
Currently smokes	0.62	0.45–0.84	**0.002**
Former smoker	1.07	0.89–1.29	0.457
Consumer price index (ref. low income)			
High income	1.14	0.96–1.35	0.139

PR: prevalence ratio; 95%CI: 95% confidence interval.

We adjusted multimorbidity for the independent variables in the multiple analysis. The outcome was statistically associated with gender, age, race/skin color, smoking habit and income in this model. Its estimate increased in all frameworks up to: 95% in women (PR _a_ = 1.95 [compared to men]; 95%CI: 1.58–2.40), 25% in individuals aged ≥ 75 years old (PR _a_ = 1.25 [compared to individuals aged ≥ 60 to 64 years old]; 95%CI: 1.01–1.60), 28% in Black people (PR _a_ = 1.28 [compared to White people]; 95%CI: 1.04–1.59), 27% in high-income individuals (PR _a_ = 1.27 [compared to low income]; 95%CI: 1.09–1.50) and 30% in former smokers (PR _a_ = 1.30 [compared to those who never smoked]; 95%CI: 1.05–1.60). Except for being a smoker, which reduced the prevalence of multimorbidity by 28% (PR _a_ = 0.72 [compared to those who never smoked]; 95%CI: 1.09–1.50) ( Table 4 ).

**Table 4:** Prevalence ratio of multimorbidity in older adults, according to independent variables in adjusted multiple. São Paulo, Brazil, 2015.

Variables	Multiple analysis		
	RP _a_	95%CI	p
Gender (ref. male)			
Female	1.95	1.58–2.40	**< 0.001**
Age (ref. 60-64 years old)			
65–69 years old	1.19	0.95–1.51	0.132
70–74 years old	1.19	0.89–1.59	0.231
≥ 75 years old	1.25	1.01–1.60	**0.042**
Race/Skin color (ref. White)			
Asian	0.98	0.66–1.44	0.922
Mixed-race	1.16	0.95–1.41	0.129
Black	1.28	1.04–1.59	**0.022**
Smoking habit (ref. never smoked)			
Currently smokes	0.72	0.53–0.99	**0.044**
Former smoker	1.30	1.05–1.60	**0.014**
Consumer price index (ref. low income)			
High income	1.27	1.09–1.50	**0.003**

PR _a_: adjusted prevalence ratio; 95%CI: 95% confidence interval.

## DISCUSSION

The prevalence of multimorbidity in older adults in the city of São Paulo, estimated at 40% (95%CI: 36.6–43.8), differs from most findings for this age group in other locations in Brazil, whose estimates ranged from 23.7% to 81.3% ^5 , 8 , 16^ . These differences are partly due to the definition and measurement criteria used in this and other studies ^3^ . Evidence shows that the heterogeneity of these methods reaches more than 90%, mainly related to the composition of the outcome ^4^ .

Despite the cutoff point of two or more chronic diseases, the multimorbidity estimate in the reviewed studies followed different previously defined lists in which some included conditions that although they did not represent illness, they were overestimated ^5^ . We chose to exclude acute conditions, risk factors or phenotypic characteristics such as high cholesterol, back pain, rhinitis, sinusitis and tendinitis. This methodological option certainly penalized the proposed models and may have affected the performed estimates.

Moreover, the definition of older adults from 50 years old instead of 60 years old, the sample design without population base and the different research scenarios may have contributed to the difference between our findings and other studies. However, the factors that changed more or less the estimates of the outcome showed consistency. This is related to the aspects usually associated with chronic diseases, such as gender, age, socioeconomic status, race/skin color and smoking habit, which are essential to study the magnitude and distribution of outcome ^6^ .

Regarding gender, the prevalence of multimorbidity in women was 95% (PR _a_ = 1.95; 95%CI: 1.58–2.40), which is higher than the estimate measured among men. Women are in a better situation compared to men in some indicators, as evidenced by the number of inmates, victims of homicides and traffic accidents, HIV infection and homeless people. The opposite is expected with multimorbidity, because this and other studies showed significant disadvantage ^5^ . Three interrelated factors can explain this: greater opportunity for diagnosis, higher prevalence of chronic diseases and survival bias.

Seeking and using health care services, which is related to access and greater perception about physical signs and clinical symptoms of illness, enabling the diagnosis; higher prevalence of chronic diseases, regardless of the age factor; and the disadvantage over the so-called surviving men, those who reach old age with health despite the high risk of death in young and old age. These are factors that may explain, with greater or lesser plausibility, why multimorbidity is more prevalent in women.

Regarding age, the prevalence of multimorbidity increases simultaneously with age, as a response pattern. The indicator increased 25% for those aged ≥ 75 years old compared to individuals aged 60 to 64 years old (PR _a_ = 1.25; 95%CI: 1.01–1.60). Age is the risk factor usually associated with the coexistence of multiple chronic conditions in older adults, which is why all reviewed multimorbidity models explore it ^7^ . The consistency of data on aging indicates a global challenge due to the increasing age structure ^22^ worldwide, especially for the Brazilian context.

The association between race/skin color and multimorbidity showed that being Black increased the prevalence of this condition by 28% (PR _a_ = 1.28; 95%CI: 1.04–1.59), compared to White individuals. The individuals’ social conditions during their lives can explain this association, increasing the vulnerability of Black people to unfavorable outcomes in old age ^23^ . Despite the controversy in certain contexts, genetic information does not explain all biological events and may have little predictive value for multimorbidity, as observed for some isolated chronic diseases, such as cardiovascular ^24^ .

A prospective analysis with 19,000 initially healthy women recorded incidences such as infarction and stroke ^24^ . The genetic information measured by scores with all potential polymorphisms associated with cardiovascular conditions did not remain an independent predictor when phenotypic aspects such as cholesterol, blood pressure, blood glucose, were not controlled. Moreover, we verified that family history had better predictive power because it reflects behavioral, environmental and social similarities among family members, which agrees with the explanatory model we used to analyze the association between race/skin color and multimorbidity, which is based on the social determination of the disease instead of genetic determinism ^24^ .

The higher prevalence of multimorbidity in women, older adults and Black people, agrees with the compared literature. Nevertheless, the high-income class showed a 27% increase in outcome higher than the consumer price index, a socioeconomic proxy used in this study (PR _a_ = 1.27; 95%CI: 1.09–1.50), compared to the low-income class. Although present in the literature ^5 , 25 , 26^ , it differs to some extent from other reviewed studies in which there is an inversely proportional pattern between income and the number of chronic conditions. That is, low-income individuals showed higher multimorbidity ^8 , 27 , 28^ .

The higher prevalence of multimorbidity in wealthier individuals may be related to at least two factors: the differences between socioeconomic indicators used in studies from different countries and in different locations of the same country and, mainly, to the organization of health care systems, whose greater or lesser influence of organizational, cultural and/or financial barriers of the health care systems worldwide limit or favor access to services and the opportunity to diagnosis. Although the Brazilian Unified Health System includes great part of the population, coverage remains unequal. This may explain why studies sometimes indicate higher estimate of the outcome among the wealthiest ^5^ , and sometimes among the poorest ^8^ .

We verified that former smokers showed higher prevalence of multimorbidity of 30% (PR _a_ = 1.30; 95%CI: 1.05–1.60), compared to those who never smoked. We cannot establish what occurred first based on the studied data, whether smoking cessation or multimorbidity. However, studies reported similar findings ^5 , 29 , 10^ and reinforce the hypothesis that the diagnosis of chronic diseases can lead to smoking cessation. The fact that the estimated outcome was significantly lower among smokers (PR _a_ = 0.72; 95%CI: 1.09–1.50) reinforces this hypothesis. Possibly because being affected by these diseases leads to greater contact with health care providers and greater exposure to educational interventions, which contribute to smoking cessation. This partially differs from what usually occurs with individuals who perceive themselves healthy or do not have any clinical diagnosis and, for this reason, support the smoking habit.

As limitations of the study, we did not advance in the usual patterns of occurrence of multimorbidity, its effect on the older adults’ functionality, on the use and costs of health care services, which future studies can better assess. Difficulties inherent to its cross-sectional nature, which do not allow to associate cause and effect, may have hindered some analyses. Furthermore, our results are based on self-reported data on illness, therefore, they were greatly or less influenced by memory bias and by the opportunity of diagnosis at health care services between different socioeconomic groups. The difference between conceptual and methodological aspects between this and other studies compromises the comparisons.

The value of this study is due to being the first to show the prevalence of multimorbidity in older adults in São Paulo, the largest city in Latin America. We emphasize the need of a conceptual effort and standardization of methodologies for comparison, especially regarding the number and chronic diseases previously defined. The consistency in the factors associated with the outcome is related to the aspects usually associated with chronic diseases, such as gender, age, socioeconomic status, race/skin color and smoking habit, and may guide studies on their population distribution. Finally, regarding the apparently contradictory data, the higher prevalence of the outcome among the wealthiest and former smokers is sometimes related to the construction of the socioeconomic indicator, sometimes to the inaccessibility to diagnosis among the poorest, and to multimorbidity presumably leading to smoking cessation.
