# Supplementary material for: Prevalence of multimorbidity in older adults in São Paulo, Brazil: a study with ISA-Capital
Source: Rev Saude Publica. 2022 Jul 21;56:69. doi: 10.11606/s1518-8787.2022056004252 (PMC9337848; doi:10.11606/s1518-8787.2022056004252)
Supplement: Translation

# Translation: Prevalência de multimorbidade em idosos em São Paulo, Brasil: um estudo com o ISA-Capital

**Authors:** Kaio Keomma, Aylene Bousquat, Chester Luiz Galvão César

**Affiliations:** I Brasil Universidade de São Paulo. Faculdade de Saúde Pública. Programa de Pós-Graduação em Saúde Pública. São Paulo, SP, Brasil; II Brasil Universidade de São Paulo. Faculdade de Saúde Pública. Departamento de Política, Gestão e Saúde. São Paulo, SP, Brasil; III Brasil Universidade de São Paulo. Faculdade de Saúde Pública. Departamento de Epidemiologia. São Paulo, SP, Brasil

**Keywords:** Idoso, Multimorbidade, Prevalência, Fatores de Risco, Inquéritos Epidemiológicos

## Abstract

**OBJETIVO:**

Estimar a prevalência de multimorbidade em idosos em São Paulo, Brasil.

**MÉTODOS:**

Estudo transversal, aninhado ao inquérito de base populacional ISA-Capital, de 2015, com sub amostra de 1.019 idosos com 60 anos ou mais. A multimorbidade foi categorizada considerando duas ou mais doenças crônicas, a partir de uma lista previamente definida. Os dados foram analisados em modelos univariado e múltiplo com a regressão de Poisson.

**RESULTADOS:**

A prevalência de multimorbidade foi de 40% (IC95% 36,6–43,8), sendo maior nas mulheres (RP _a_ = 1,95 [comparado com homens]; IC95% 1,58–2,40), nos indivíduos com 75 anos ou mais (RP _a_ = 1,25 [comparado com indivíduos de 60 a 64 anos]; IC95% 1,01–1,60), nos pretos (RP _a_ = 1,28 [comparado com brancos]; IC95% 1,04–1,59), nas pessoas de alta renda (RP _a_ = 1,27 [comparado com baixa renda]; IC95% 1,09–1,50) e nos ex-fumantes (RP _a_ = 1,30 [comparado com quem nunca fumou]; IC95% 1,05–1,60) e menor nos que se declararam fumantes (RP _a_ = 0,72 [comparado com quem nunca fumou]; IC95% 1,09–1,50).

**CONCLUSÕES:**

A prevalência de multimorbidade foi inferior à reportada na maioria dos estudos revisados, mas há consistência sobre sua associação com sexo, idade, cor da pele, tabagismo e nível socioeconômico. A padronização de critérios conceituais e metodológicos para a sua estimação é um desafio para mitigar problemas no planejamento e gestão de sistemas de saúde para populações cada vez mais envelhecidas.

## INTRODUÇÃO

Multimorbidade é a coexistência de múltiplas doenças crônicas em um mesmo indivíduo ^1^ . É amplamente reportada considerando duas afecções ^2^ , mas não há consenso em sua conceituação e tampouco sobre os métodos mais adequados para estimá-la – que incluem de contagens simples por indivíduo a sistemas sofisticados de classificação para a medição da carga de morbidade ^3^ , o que resulta em uma ampla variabilidade de estimativas e associações ^4^ .

A multimorbidade é um importante problema no quadro sanitário nacional e internacional ^5^ . Achados dão conta de que a sua prevalência alcança mais da metade dos idosos ^6^ , sendo maior em mulheres ^7^ , indivíduos mais pobres e moradores de zona urbana ^8^ , pretos ^9^ e ex-fumantes ^10^ , com importante impacto na mortalidade ^11 , 12^ . Isso tornou-se mais evidente, por exemplo, com a covid-19 (SARS-Cov-2), em que pacientes crônicos e com multimorbidade representavam até 72% dos pacientes internados em unidades de terapia intensiva em algumas localidades ^13^ . Há maior vulnerabilidade desses indivíduos durante a pandemia ^14^ e o desconhecimento deste indicador em várias localidades e o maior alinhamento dos serviços para condições agudas, restrição às consultas e procedimentos eletivos, dentre outras, pode contribuir para isso.

A multimorbidade tem repercutido de forma relevante em indicadores como hospitalizações sucessivas, internações prolongadas, polifarmácia, uso simultâneo de vários serviços de diferentes níveis de densidade tecnológica, custo assistencial e principalmente na coordenação do cuidado, porquanto requer uma multiplicidade de pessoas e serviços envolvidos na provisão do cuidado ^15^ . Compreender essas e outras questões é central para o sistema de saúde e passa necessariamente pelo conhecimento sobre a sua magnitude e distribuição. Contudo, pesquisas que estimaram a prevalência de multimorbidade são incipientes no Brasil, especialmente com amostras representativas ^5 , 8 , 16^ .

O presente artigo pretende contribuir para preencher parte da lacuna em estudos sobre multimorbidade de base populacional na literatura brasileira, oferecendo mais subsídio à crítica e ao debate, com a disponibilização de evidências para a comunidade de pesquisadores, gestores e a sociedade. Diante disso, o objetivo deste artigo é analisar a prevalência de multimorbidade em idosos na cidade de São Paulo, Brasil.

## MÉTODOS

Estudo transversal, com 1.019 idosos com 60 anos ou mais participantes do inquérito de saúde ISA-Capital, pesquisa de base populacional da cidade de São Paulo, Brasil, realizada em 2015.

A amostragem do estudo original ocorreu por metodologia complexa e probabilística, com sorteios de setores censitários e domicílios. Foram considerados dois domínios, geográfico e demográfico, contemplando, respectivamente, as coordenadorias regionais de saúde (Centro-Oeste, Leste, Norte, Sudeste e Sul) e diferentes grupos etários (adolescentes 12 a 19 anos de idade, homens adultos 20 a 59 anos, mulheres adultas 20 a 59 anos e homens e mulheres de 60 anos ou mais). Considerou-se a expressão algébrica $n\;=\;p\;.\;\left(1-p\right)/{\left(d/z\right)}^{2}deff$ . *deff* , na qual: *n* é o tamanho da amostra, *p* é o parâmetro a ser estimado, *z* = 1,96 é o valor na curva normal reduzida referente ao nível de confiança de 95% dos intervalos de confiança, *d* é erro e *deff* é o efeito do delineamento ^20^ .

Os participantes foram recrutados considerando os critérios de inclusão: estar dentro das faixas-etárias contempladas, residir na zona urbana e em domicílios particulares de forma permanente. Foram excluídas as pessoas em situação de rua ou residentes em instituições. A taxa de não-resposta em virtude de domicílios vagos, fechados, com recusa ou com morador incapacitado de responder foi observada. Para que um número mínimo de entrevistas fosse alcançado na presença de potencial perda, foi planejada a inclusão de uma amostra maior do que o necessário. Com isso, o número almejado de entrevistas com os participantes foi atingido ^20^ .

As variáveis consideradas incluíram: sexo (masculino/feminino); idade em anos completos (60–64, 65–69, 70–74, 75 anos ou mais); cor da pele (branca, preta, parda e amarela); índice de bens, que foi considerado um *proxy* de nível socioeconômico, tendo como base o Critério de Classificação Econômica Brasil (CCEB), dicotomizado em categorias alta renda (congregando estratos originalmente classificados como A1, A2 e B1) e baixa renda (para estratos B2, C1 e C2, D, E); tabagismo (nunca fumou, ex-fumante, fumante); e multimorbidade, aferida por meio da contagem de duas ou mais morbidades autorreferidas a partir de uma lista previamente elaborada ^7^ .

Para composição da multimorbidade, foram consideradas 10 doenças crônicas disponíveis no estudo de base: hipertensão arterial sistêmica, diabetes mellitus tipo 2, osteoporose, artrite/reumatismo, acidente vascular encefálico, infarto agudo do miocárdio, doença pulmonar obstrutiva crônica (DPOC), neoplasia, alzheimer e parkinson. Foram excluídas condições que representam afecções agudas, fatores de risco ou características fenotípicas como colesterol alto, dor nas costas, rinite, sinusite e tendinite. Embora não haja apenas uma definição dessa variável que possa ser amplamente aceita ^21^ , principalmente no que se refere ao número e as condições incluídas, a estratégia utilizada é consistente com a literatura revisada, porquanto contempla múltiplos sistemas corporais e agrupamentos em torno de distúrbios cardiovasculares, metabólicos e musculoesqueléticos ^7^ .

A análise dos dados foi operacionalizada com o módulo *Survey* do programa Stata, versão 14.0, para dados obtidos em amostras complexas. Estimou-se a prevalência de multimorbidade, geral e segundo as variáveis sexo, idade, cor da pele, tabagismo e índice de bens, incluindo o cálculo de proporção de ocorrência (%), intervalo de confiança de 95% (IC95%) e valor de p pelo teste de qui-quadrado de Pearson. Modelos univariado (não ajustado) e múltiplo (ajustado) foram propostos para avaliar a variação na prevalência de multimorbidade, variável desfecho e as demais variáveis independentes, com a regressão de Poisson. Foram obtidas as razões de prevalência bruta (RP) e ajustada (RP _a_ ) e IC95%. Associações que apresentaram IC95% sem incluir a nulidade (RP = 1,00) e valor de p < 0,05 foram consideradas estatisticamente significativas.

Para fins de inferência estatística, todas as análises realizadas – obtenção de proporções, intervalos de confiança e testes – foram ponderadas com o peso final dos participantes, calculado considerando: (1) Peso de delineamento, que leva em conta as frações de amostragem das duas etapas de sorteio, a de setor censitário e a de domicílio; (2) Ajuste de não resposta, que leva em conta as taxas de resposta observadas; (3) Pós-estratificação, que ajusta a distribuição da amostra por sexo, faixa etária local de residência, de acordo com a distribuição da população do cenário de estudo estimada para o ano da pesquisa.

O ISA-SP foi aprovado pelo comitê de ética e pesquisa da Faculdade de Saúde Pública da Universidade de São Paulo e todos os preceitos éticos e legais foram observados.

## RESULTADOS

Os participantes do estudo eram predominantemente mulheres (59,7%), a média de idade foi de 67,7 anos (DP = 7,7), um quarto estava com 75 anos ou mais (25,2%), a maioria referiu cor de pele branca (59,8%), baixa renda (81%) e nunca ter fumado (63,4%) ( Tabela 1 ).

**Tabela 1:** Caracterização dos participantes do estudo. São Paulo, Brasil, 2015.

Variáveis	n (%) ^a^
Sexo	
Feminino	632 (59,7)
Masculino	387 (40,3)
Idade	
60–64 anos	310 (30,9)
65–69 anos	263 (26,2)
70–74 anos	185 (17,5)
≥ 75 anos	261 (25,2)
Cor da pele	
Preta	134 (11,9)
Parda	263 (23,7)
Branca	571 (59,8)
Amarela	39 (4,3)
Tabagismo	
Ex-fumante	249 (24,5)
Nunca fumou	644 (63,4)
Fumante	124 (12,3)
Índice de bens	
A (alta renda)	176 (23,6)
B (baixa renda)	749 (76,4)

^a^ Número de entrevistados sem ponderação.

A prevalência geral de multimorbidade foi estimada em 40% (IC95% 36,6–43,8), sendo significativamente maior entre as mulheres (49,1%; IC95% 44,7–54,0; p < 0,001), na faixa-etária de 75 anos ou mais (45,1%; IC95% 37,6–54,0; p = 0,036) e em ex-fumantes (44,3%; IC95% 38,2–51,5; p = 0,002) ( Tabela 2 ).

**Tabela 2:** Prevalência de multimorbidade em idosos, segundo variáveis independentes. São Paulo, Brasil, 2015.

Variáveis	(IC95%)	p
Sexo		**< 0,001**
Feminino	49,1 (44,7–54,0)	
Masculino	26,5 (22,1–31,8)	
Idade		**0,036**
60–64 anos	34,4 (29,0–40,9)	
65–69 anos	40,5 (34,7–47,2)	
70–74 anos	42,0 (34,6–51,1)	
≥ 75	45,1 (37,6 – 54,0)	
Cor da pele		0,591
Preta	46,3 (38,5–55,8)	
Parda	41,3 (34,8–48,9)	
Branca	38,5 (33,9–43,6)	
Amarela	37,8 (24,0–59,4)	
Tabagismo		**0,002**
Ex-fumante	44,3 (38,2–51,5)	
Nunca fumou	41,3 (36,9–46,1)	
Fumante	25,6 (18,9–34,4)	
Índice de bens		0,149
Alta renda	43,5 (36,8–51,4)	
Baixa renda	38,1 (34,5–42,2)	

IC95%: intervalo de confiança de 95%.

Na análise univariada, na qual a multimorbidade foi analisada sem ajuste para as variáveis independentes, ser mulher (RP = 1,85 [comparado com homens]; IC95% 1,52–2,25), ter 75 anos ou mais (RP = 1,30 [comparado com indivíduos de 60 a 64 anos]; IC95% 1,01–2,68) e ser fumante (RP = 0,62; IC95% 0,45–0,84) apresentaram associação significativa. Com exceção desta última condição que reduziu a multimorbidade em 38%, os dois primeiros a elevaram em 85% e 30%, respectivamente ( Tabela 3 ).

**Tabela 3:** Razão de prevalência de multimorbidade em idosos, segundo variáveis independentes em modelo univariado não-ajustado. São Paulo, Brasil, 2015.

Variáveis	Análise univariada		
	RP	IC95%	p
Sexo (ref. masculino)			
Feminino	1,85	1,52–2,25	**< 0,001**
Idade (ref. 60–64 anos)			
65–69 anos	1,18	0,94–1,46	0,147
70–74 anos	1,22	0,93–1,59	0,137
≥ 75 anos	1,30	1,01–1,68	**0,035**
Cor da pele (ref. branca)			
Amarela	0,98	0,63–1,53	0,938
Parda	1,07	0,87–1,32	0,519
Preta	1,20	0,97–1,48	0,078
Tabagismo (ref. nunca fumou)			
Fuma atualmente	0,62	0,45–0,84	**0,002**
Ex-fumante	1,07	0,89–1,29	0,457
Índice de bens (ref. baixa renda)			
Alta renda	1,14	0,96–1,35	0,139

RP: razão de prevalência; IC95%: intervalo de confiança de 95%.

Na análise múltipla a multimorbidade foi ajustada para o conjunto de variáveis independentes. Nesse modelo, o desfecho esteve estatisticamente associado a sexo, idade, cor de pele, tabagismo e renda. A sua estimativa aumentou em todos os recortes considerados, a patamares de: 95% em mulheres (RP _a_ = 1,95 [comparado com homens]; IC95% 1,58–2,40), 25% em indivíduos de 75 anos ou mais (RP _a_ = 1,25 [comparado com indivíduos de 60 a 64 anos]; IC95% 1,01–1,60), 28% em pretos (RP _a_ = 1,28 [comparado com brancos]; IC95% 1,04–1,59), 27% em indivíduos de alta renda (RP _a_ = 1,27 [comparado com baixa renda]; IC95% 1,09–1,50) e 30% em ex-fumantes (RP _a_ = 1,30 [comparado com quem nunca fumou]; IC95% 1,05–1,60). Exceção se fez a ser fumante, aspecto que reduziu a prevalência de multimorbidade em 28% (RP _a_ = 0,72 [comparado com quem nunca fumou]; IC95% 1,09–1,50) ( Tabela 4 ).

**Tabela 4:** Razão de prevalência de multimorbidade em idosos, segundo variáveis independentes em múltiplo ajustado. São Paulo, Brasil, 2015.

Variáveis	Análise múltipla		
	RP _a_	IC95%	p
Sexo (ref. masculino)			
Feminino	1,95	1,58–2,40	**< 0,001**
Idade (ref. 60-64 anos)			
65–69 anos	1,19	0,95–1,51	0,132
70–74 anos	1,19	0,89–1,59	0,231
≥ 75 anos	1,25	1,01–1,60	**0,042**
Cor da pele (ref. branca)			
Amarela	0,98	0,66–1,44	0,922
Parda	1,16	0,95–1,41	0,129
Preta	1,28	1,04–1,59	**0,022**
Tabagismo (ref. nunca fumou)			
Fuma atualmente	0,72	0,53–0,99	**0,044**
Ex-fumante	1,30	1,05–1,60	**0,014**
Índice de bens (ref. baixa renda)			
Alta renda	1,27	1,09–1,50	**0,003**

RP _a_: razão de prevalência ajustada; IC95%: intervalo de confiança de 95%.

## DISCUSSÃO

A prevalência de multimorbidade em idosos na cidade de São Paulo, estimada em 40% (IC95% 36,6–43,8), não é similar à maioria dos achados para essa faixa etária encontrados em outras localidades do Brasil, cujas estimativas variaram enormemente de 23,7% a 81,3% ^5 , 8 , 16^ . Essas diferenças decorrem, em parte, dos critérios de definição e mensuração, utilizados neste e em outros estudos ^3^ . Evidências apontam que a heterogeneidade desses métodos alcança patamares superiores a 90%, principalmente relacionadas à composição do desfecho ^4^ .

Sobre o exposto, embora com ponto de corte de duas ou mais doenças crônicas, a estimação da multimorbidade nos estudos revisados foi orientada por diferentes listagens previamente definidas, em algumas das quais foram incluídas condições que embora não representem adoecimento, a superestimaram ^5^ . Nesta pesquisa, contudo, optou-se por excluir condições que representam afecções agudas, fatores de risco ou características fenotípicas como colesterol alto, dor nas costas, rinite, sinusite e tendinite. Essa opção metodológica decerto penalizou os modelos propostos e pode ter impactado as estimações realizadas.

Ademais, a definição de idosos a partir de 50 anos e não 60 anos, o delineamento de amostras sem base populacional e os distintos cenários de pesquisa também podem ter contribuído, ainda que presumivelmente, para a diferença entre os achados deste e dos demais estudos. Houve, porém, certa consistência nos fatores que alteraram para mais ou para menos as estimações do desfecho. Isso decerto está relacionado aos aspectos mais comumente associados às próprias doenças crônicas como o sexo, a idade, o nível socioeconômico, a cor da pele e o tabagismo que possuem importância para o estudo da magnitude e distribuição do desfecho ^6^ .

Em relação ao sexo verificou-se que a prevalência de multimorbidade em mulheres foi 95% (RP _a_ = 1,95; IC95% 1,58–2,40) superior quando comparado à estimativa aferida entres os homens. É verdade que, para alguns indicadores, indivíduos do sexo feminino estão em melhor situação quando comparados aos do sexo masculino, como fica evidente em números de encarcerados, vítimas de homicídios e de acidentes de trânsito, infecção pelo HIV e pessoas em situação de rua. Com a multimorbidade espera-se o oposto, porquanto neste e em outros estudos demonstrou-se expressiva desvantagem ^5^ . Isso pode ser explicado por ao menos três fatores interrelacionados: maior oportunidade de diagnóstico, maior prevalência de doenças crônicas e viés de sobrevida.

Procurar e utilizar mais a rede de serviços de saúde, o que está relacionado com o acesso, mas também com a maior percepção sobre os sinais físicos e sintomas clínicos de adoecimento, gerando maior oportunidade de diagnóstico; maior prevalência de doenças crônicas, independentemente do fator etário ora referido; e, finalmente, a desvantagem sobre os chamados homens sobreviventes, aqueles que a despeito do alto risco de morte na idade jovem e adulta alcançam a velhice com mais saúde; tudo isso são fatores que podem explicar, com maior ou menor plausibilidade, o porquê de a multimorbidade ser mais prevalente em mulheres, conforme foi verificado.

Para a idade, demonstrou-se que a prevalência de multimorbidade aumenta à medida em que a faixa etária também se eleva, como um padrão de resposta. Comparado aos indivíduos de 60 a 64 anos, aqueles com 75 anos ou mais, teve o indicador elevado em 25% (RP _a_ = 1,25; IC95% 1,01–1,60). A idade é o fator de risco mais comumente associado à coexistência de múltiplas condições crônicas em idosos, motivo pelo qual é explorado em todos os modelos de multimorbidade revisados ^7^ . Decerto a consistência de dados que tem se consolidado sobre o envelhecimento aponta para um importante desafio global dada a estrutura etária cada vez mais envelhecida ^22^ no mundo, sobretudo para a realidade brasileira.

De outra associação investigada, a cor de pele e a multimorbidade, depreendeu-se que ser preto elevou a prevalência desta condição em 28% (RP _a_ = 1,28; IC95% 1,04–1,59); em relação aos indivíduos brancos. Acredita-se que essa associação pode ser apropriadamente explicada pelas condições sociais a que estão expostos aqueles indivíduos ao longo da vida, aumentando a vulnerabilidade dos pretos a desfechos desfavoráveis na velhice ^23^ . Embora possa soar controverso em certos contextos, acredita-se que informações genéticas não explicam todos os eventos biológicos e podem ter pouco valor preditivo para a multimorbidade, tal como se observa para algumas doenças crônicas isoladas como as cardiovasculares ^24^ .

Em análise prospectiva com 19 mil mulheres inicialmente saudáveis, registrou-se incidências de condições como infarto e AVC ^24^ . A informação genética aferida por escores com todos os potenciais polimorfismos previamente associados com condições cardiovasculares não permaneceu preditora independente, quando não controlado aspectos fenotípicos como colesterol, pressão arterial, glicemia. Ademais, verificou-se que história familiar teve melhor poder preditor, porquanto reflete similaridades comportamentais, ambientais e sociais entre familiares, o que vai ao encontro do modelo explicativo utilizado neste estudo para analisar a associação entre cor da pele e multimorbidade, que se assenta na determinação social da doença e não no determinismo genético ^24^ .

A maior prevalência de multimorbidade em mulheres, indivíduos com faixas-etárias mais elevadas e pretos, como se demonstrou, são pontos mais consensuais com literatura comparada. Não obstante, o aumento do desfecho tanto quanto maior o índice de bens, um *proxy* para nível socioeconômico utilizado neste estudo, em patamares de 27% (RP _a_ = 1,27; IC95% 1,09–1,50) para a classe alta renda comparada com a de baixa renda, embora presente na literatura ^5 , 25 , 26^ , diverge em certa medida de outros estudos revisados, nos quais há um padrão inversamente proporcional entre a renda e o número de condições crônicas. Isto é, indivíduos economicamente mais desfavoráveis apresentaram maior multimorbidade ^8 , 27 , 28^ .

Crê-se que a maior prevalência de multimorbidade em indivíduos mais ricos pode estar relacionado a pelo menos dois fatores: às diferenças entre os indicadores socioeconômicos utilizados em estudos de diferentes países e em distintas localidades de um mesmo país e, principalmente, às formas de organização dos sistemas de saúde, cuja maior ou menor influência de barreiras organizacionais, sociais, culturais e/ou financeiras que caracteriza os diferentes sistemas de saúde no mundo, limitam ou favorecem o acesso aos serviços e a oportunidade de diagnóstico. A despeito do sistema público de saúde do Brasil incluir amplos segmentos da população a cobertura ainda é sobremaneira desigual. Provavelmente por isso os estudos ora apontem para maior estimativa do desfecho entre os mais ricos ^5^ , ora para entre os mais pobres ^8^ .

Finalmente, verificou-se que ex-fumantes apresentaram uma prevalência maior de multimorbidade em um patamar de 30% (RP _a_ = 1,30; IC95% 1,05–1,60), em relação aos que nunca fumaram. Não é possível, porém, com os dados aqui aferidos, estabelecer o que ocorreu primeiro, se a cessação do tabagismo ou a multimorbidade. Porém, achados semelhantes têm sido reportados ^5 , 29 , 10^ e reforça a hipótese segundo a qual o diagnóstico de doenças crônicas pode levar a cessação do tabagismo. O fato de a estimativa do desfecho ser significativamente menor entre os que se declararam fumantes (RP _a_ = 0,72; IC95% 1,09–1,50) reforça essa hipótese. Isso porque acredita-se que ser acometido por aquelas afecções levem as pessoas a um maior contato com profissionais de saúde e a uma maior exposição a intervenções educacionais, contribuindo para o abandono da prática. Isso difere, em parte, do que normalmente ocorre com indivíduos que se autopercebem saudáveis ou não possuem algum diagnóstico clínico e que, exatamente por isso, sustentam o tabagismo.

Como limitações do estudo, não se avançou nos padrões mais comuns de ocorrência da multimorbidade, no seu impacto na funcionalidade do idoso, na utilização e nos custos de serviços de saúde, aspectos que poderão ser investigados com maior abrangência em estudos futuros. Dificuldades inerentes à sua natureza transversal, que não permitem realizar associações de causa e efeito, podem ter prejudicado algumas análises. Ainda, como os resultados aqui apresentados são baseados em dados autorreferidos sobre adoecimento, em maior ou menor grau foram influenciados pelo viés de memória e certamente pela oportunidade de diagnóstico nos serviços de saúde entre grupos de diferentes níveis socioeconômicos. Finalmente, a diferença entre aspectos conceituais e metodológicos entre este e os demais estudos da literatura comprometem as comparações que devem ser realizadas de forma parcimoniosa.

O valor deste estudo, porém, está no ineditismo em apresentar a prevalência de multimorbidade em idosos em São Paulo, a maior cidade da América Latina. Enfatizou-se a necessidade de um esforço conceitual e de padronização de metodologias para permitir a comparação, principalmente em relação ao número e às doenças crônicas previamente definidas. A consistência nos fatores associados ao desfecho decerto está relacionada aos aspectos mais comumente associados às próprias doenças crônicas, como sexo, idade, nível socioeconômico, cor da pele e tabagismo, e podem guiar processos de estudo sobre a sua distribuição populacional. Finalmente, sobre dados aparentemente contraditórios apresentados, a despeito da maior prevalência do desfecho entre os mais ricos e ex-fumantes, reforça-se que isso está relacionado ora à construção do indicador socioeconômico, ora ao difícil acesso a oportunidade diagnóstica entre os mais pobres, de um lado, e ao fato de que a multimorbidade presumivelmente leva a cessação do tabagismo, do outro lado.
